# Supplementary material for: Lipopolymers as the Basis of Non-Viral Delivery of Therapeutic siRNA Nanoparticles in a Leukemia (MOLM-13) Model
Source: Biomolecules. 2025 Jan 13;15(1):115. doi: 10.3390/biom15010115 (PMC11763671; doi:10.3390/biom15010115)
Supplement: Supplementary file 1 [file biomolecules-15-00115-s001.zip › biomolecules-3272829-supplementary.pdf]

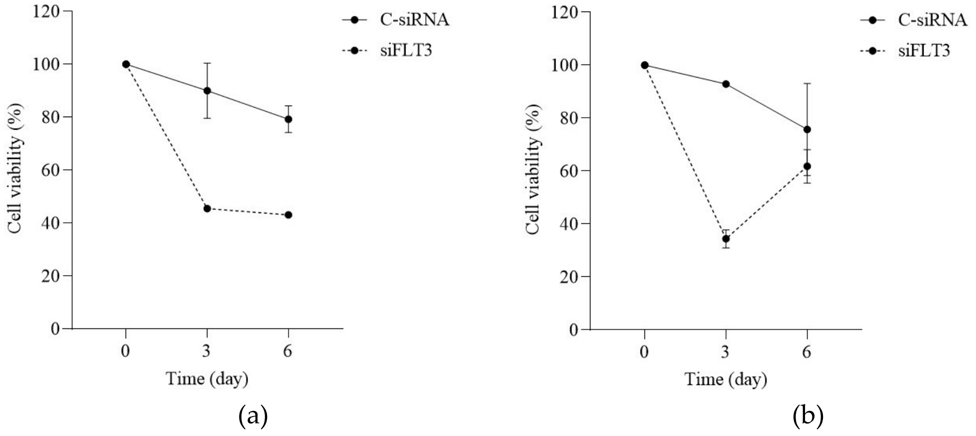

Figure S1. Viability of MOLM-13 cells treated with polymer/siRNA complexes as a function of time. MOLM-13 cells that were treated with polymer/siRNA complexes (60 nM siRNA formulated with 6.0:1.0 (w/w) polymer/siRNA ratio) and were determined by the MTT assay. Percent cell viability was determined at 3- and 6-days post-treatment with C-siRNA and siFLT3 complexes formulated with **(a)** Prime-Fect, **(b)** PEI1.2k-PHPA-Lin9. The results are presented as the mean ± SD viability relative to untreated cells, which are considered as 100% viability (n = 3). Note that Prime-Fect treated cells gave more durable inhibition of cell growth.

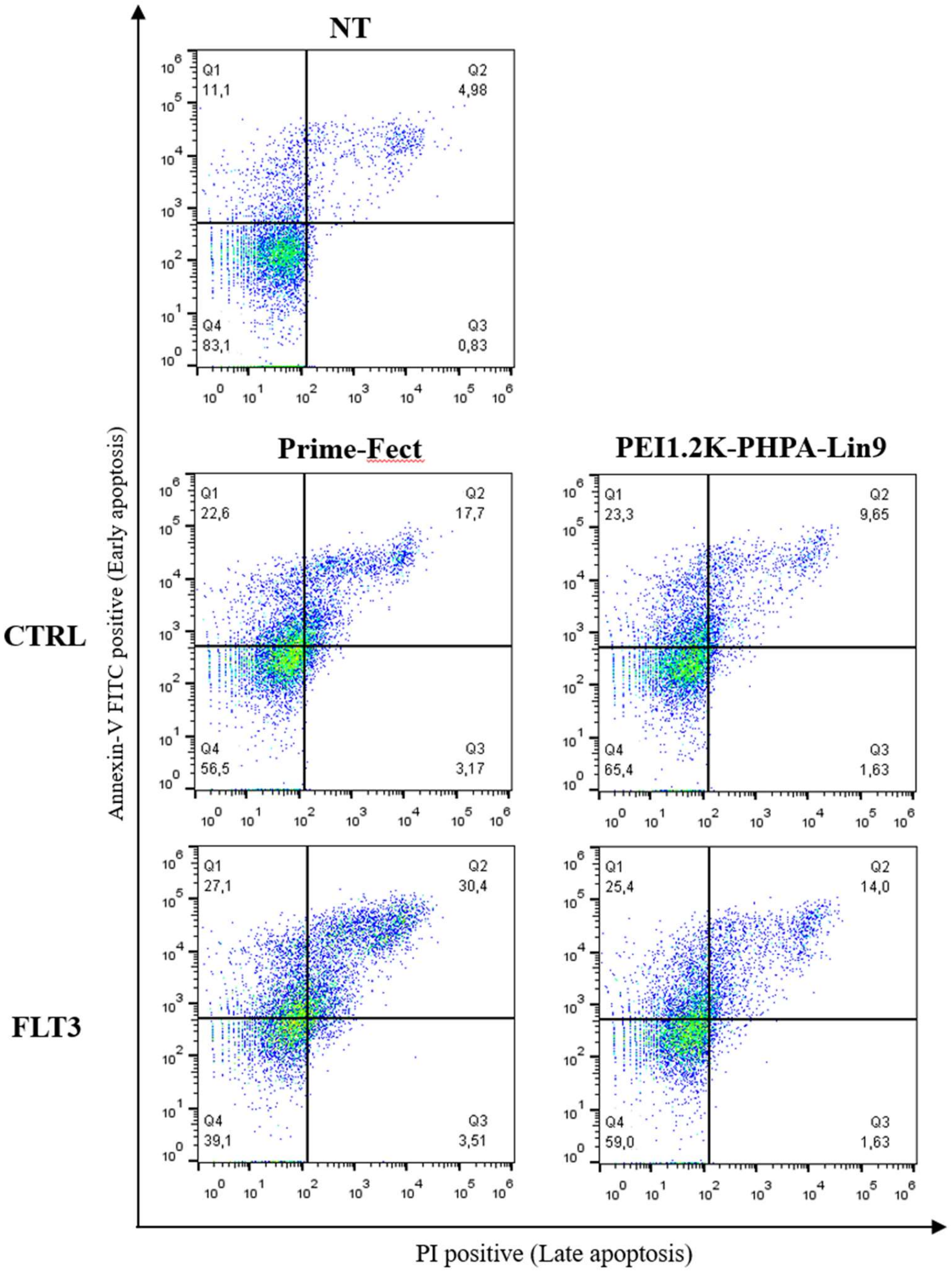

Figure S2. Representative histograms for the apoptosis assay from the flow cytometry. **Top panel:** Non-treated (NT) cells. **Middle row:** Cells treated with control siRNA complexes. **Bottom row:** Cells treated with FLT3 siRNA complexes. Left panels in the middle and bottom rows are related to Prime-Fect complexes whereas right panels refer to PEI1.2-PHPA-Lin9 complexes.

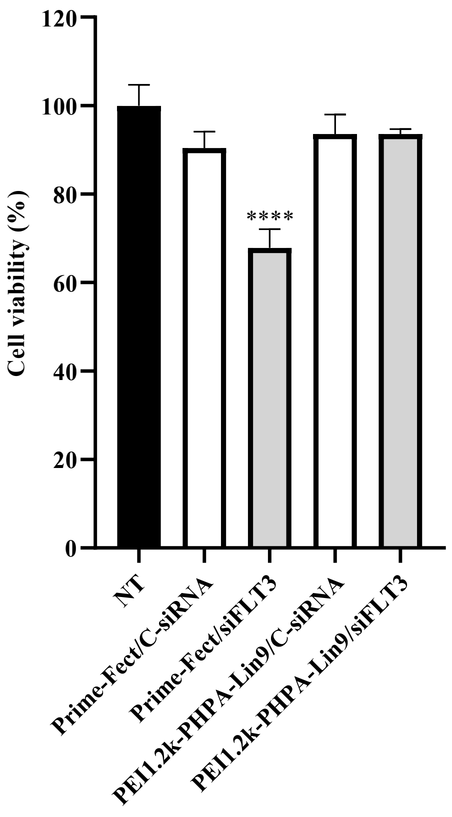

Figure S3. Response to siRNA complexes in Luc+ MOLM-13 cells in vitro. Percent cell viability was measured by the MTT assay after 72 hours of a single treatment at an siRNA concentration of 60 nM. The siRNA was either control (scrambled) or FLT3-specific, delivered by Prime-Fect or PEI1.2k-PHPA-Lin9. Results are shown as mean + SD of quadruplicate wells and expressed with respect to non-treated (NT) cells (taken as 100%). Significance was analyzed by one-way ANOVA with multiple comparison test; **** $p \leq 0.0001$ versus control siRNA.
